# A case of hemothorax due to traumatic bleeding managed effectively by Sonoclot®-guided blood transfusion

**DOI:** 10.1186/s40981-016-0042-9

**Published:** 2016-08-02

**Authors:** Mitsutaka Edanaga, Tomoe Hoshi, Ryu Azumaguchi, Michiaki Yamakage

**Affiliations:** 1 Department of Anesthesiology, Sapporo Medical University School of Medicine, South 1, West 16, Chuo-ku, Sapporo, Hokkaido 060-8543 Japan; 2 Department of Anesthesia, Japanese Red Cross Kitami Hospital, Kitami, Japan

**Keywords:** Traumatic bleeding, Conventional blood coagulation test, Sonoclot® analyzer

## Abstract

A 71-year-old woman was transported to our hospital due to traumatic bleeding, and an operation was immediately performed for achieving hemostasis. We decided to perform Sonoclot®-guided blood transfusion. When Sonoclot signatures had returned normal values, further bleeding did not occur. We experienced the first case of traumatic bleeding managed effectively by using Sonoclot. We suggest that a Sonoclot analyzer may be useful for the management of severe coagulopathy due to traumatic bleeding like ROTEM and TEG.

## Background

Appropriate management and treatment of fluid and blood transfusion resuscitation for a case of massive bleeding is difficult. The amount and type of transfusion must usually be considered to estimate the degree of surgical site bleeding. Sonoclot® analyzer is a device for point-of-care monitoring of blood coagulation [1]. It can mainly show three signatures [2]: activated coagulation time (SonACT), degree of fibrin formation (clot rate (CR)), and ability of the clot retraction (platelet function (PF)), all of which can be measured within about 20 min [3]. We could manage effectively for a case of hemothorax due to traumatic bleeding by using Sonoclot.

## Case presentation

A 71-year-old woman (weight, 86 kg; height, 158 cm; blood type, type A) was transported to another hospital for hemodynamic unstability due to trauma. She was suspected of having right hemothorax at that time. Thus, she was immediately transported to the Department of Emergency Medicine, Advanced Critical Care and Emergency Center Care Unit in Sapporo Medical University Hospital. Although she had severe hypotension (about 40 mmHg) during the transportation by ambulance, blood pressure at admission to our hospital was 74/45 mmHg, heart rate was 86 bpm, arterial saturation by oxygen inhalation (10 L/min mask) was 95 %, and Glasgow Coma Scale was 12. She was intubated and central venous catheterization was performed. She was then immediately admitted to operating room. Laboratory data before admission to the operating room showed severe coagulation disorders as follows (Table 1): hemoglobin was 15.9 g/dL, platelet count was 26,000/μL, international normalized ratio of prothrombin time (PT-INR) was 1.6, activated partial thromboplastin time (APTT) was 59.3 s, and fibrinogen was 65 mg/dL. Blood pressure at admission to operating room was 66/36 mmHg, heart rate was 77 bpm, arterial saturation was 100 %, and body temperature was 36.0°. During the operation, the amount of bleeding after 2 h exceeded 7000 mL. Thus, we considered the necessity for point-of-care monitoring of blood coagulation for appropriate transfusion. We decided to perform Sonoclot-guided transfusion. The first data (Table 2) were obtained at 2 h after starting the operation. The first data were as follows (point 1): SonACT was 581 s, CR was 1.5, and PF was 0.5. Thus, we continued to perform massive transfusion. The amounts of red blood cells (RBC), fresh frozen products (FFP), and platelets at that time were 28, 28, and 20 units, respectively. Although the degree of bleeding gradually decreased, the amount of bleeding reached 9000 mL. The second Sonoclot signature (point 2) showed that SonACT was 258 s, CR was 6.5, and PF was 0.5. Thus, we mainly performed transfusion of FFP and platelets. We measured the Sonoclot signature again (point 3) since bleeding from the surgical site had almost stopped. SonACT was 193 s, CR was 11.3 (normal range), and PF was 3.2 (normal range). We therefore did not perform additional transfusion. The bleeding was diminished, and the operation was soon finished. Total bleeding was 9680 mL, and total amounts of RBC, FFP, and platelets transfused were 36, 46, and 40 units, respectively. The cell-salvaged blood was also transfused 2750 mL. Laboratory data after the operation were as follows: hemoglobin was 6.7 g/dL, platelet count was 74,000/μL, PT-INR was 1.31, APTT was 52.5 s, and fibrinogen was 136 mg/dL. Blood pressure at operation finished was 116/59 mmHg, heart rate was 77 bpm, arterial saturation was 100 %, and body temperature was 35.6°. She was transported to the Advanced Critical Care and Emergency Center Care Unit, but further bleeding did not occur and she gradually recovered.

**Table 1:** Change in data obtained by conventional tests during the operation

	PT-INR normal <1.16	APTT normal <37 s	Fibrinogen normal >200 mg/dL	Platelet count normal >15 × 10^4^/μL
Before operation	1.6	59.3	65	2.6 × 10^4^
After operation	1.31	52.5	136	7.4 × 10^4^

Although conventional blood coagulation tests before the operation showed severe coagulation disorders, there were slightly improvements after operation

**Table 2:** Changes in three signatures of Sonoclot® analyzer during the operation

	SonACT normal 100–155 s	Clot rate (CR) normal 9–35	Platelet function (PF) normal >1.5
Point 1	581	1.5	0.5
Point 2	258	6.5	0.5
Point 3	193	11.3	3.2

Although all signatures of the Sonoclot analyzer, which is a point-of-care monitor of clotting factor and platelet function, showed severe coagulopathy (points 1 and 2), CR and PF, but not SonACT, were in normal ranges after the bleeding had stopped (point 3)

### Discussion

We experienced a typical case of hemorrhagic shock due to traumatic bleeding, and the first clinical case of traumatic patient managed effectively by using Sonoclot. Appropriate management and treatment of fluid and blood transfusion resuscitation for a case of massive bleeding as in our case is difficult. In the circumstances, severe coagulopathy inevitably occur. The amount and type of blood must be considered for estimating the degree of surgical site bleeding. However, we also think that a useful and appropriate parameter or guideline to support the clinical decision for blood transfusion in a case of massive hemorrhage is necessary. For example, arterial blood gas analysis can be performed quickly. But arterial blood gas analysis is useful as a guide for RBC transfusion. Also, conventional blood coagulation tests for PT-INR, APTT, and fibrinogen usually take much time for the indication of FFP or platelet transfusion.

European guidelines for the management of bleeding following major trauma were published in 2007 [4]. According to the guidelines, point-of care monitoring of blood coagulation such as thrombelastometry might be useful for more accurate targeting of therapy [5]. However, there are no guidelines for the treatment of traumatic bleeding in Japan. Thus, we have to manage and treat massive bleeding based on our own clinical decision. Although it was thought that the viscoelastic device as ROTEM and TEG was useful for trauma patients [6], no data on clinical effectiveness of Sonoclot for traumatic bleeding was reported. Sonoclot analyzer is always disposed in our operating room. And we found that Sonoclot was useful as a guide for blood transfusion during cardiac surgery and massive bleeding in a clinical situation and in our clinical study (not yet published). Thus, we thought that a Sonoclot analyzer is very useful for management of coagulopathy and used a Sonoclot analyzer in our case. The main benefits of this device are as follows. Firstly, when we use the analyzer, we can evaluate not only blood clotting function as the degree of fibrin gel formation but also platelet function as clot retraction. Secondly, data can be obtained from the Sonoclot analyzer after only 15 to 20 min. Thirdly, Sonoclot analyzer can perform measurements using whole blood. While, the disadvantage of Sonoclot compared to ROTEM is that we cannot evaluate the exact cause for severe coagulopathy, for example, intrinsic factor, extrinsic factor, or fibrinogen. Therefore, when we would like to check an exact cause for it, we should consider using not only Sonoclot but also other device like ROTEM.

By the way, measurements of PT-INR and APTT are performed using blood contained no platelet. Platelet and coagulation factor are greatly related to the intracellular collect blood coagulation system [7]. Therefore, we think that the values of PT-INR and APTT are not an appropriate signature for blood coagulation. In the European guideline mentioned above [5], it was reported that PT-INR and APTT alone should not be used to guide hemostatic therapy. In fact, conventional blood coagulation tests after the operation showed some coagulopathy. The final SonACT and postoperative APTT were slightly prolonged. According to the past report [8], the cell-salvaged transfusion may cause coagulopathy. The cell-salvaged blood was transfused 2750 ml during the operation. Although we should consider the amount of cell-salvaged blood, CR and PF of Sonoclot signature was normal and postoperative further bleeding did not occur. Therefore, our results indicate that the value of Sonoclot may be reliable rather than PT-INR and APTT.

## Conclusions

We experienced the first case of traumatic bleeding managed effectively by using Sonoclot. Although the further data is necessary, we suggest that the Sonoclot analyzer may be useful for the management of severe coagulopathy due to not only traumatic bleeding but also massive bleeding of emergent situation.
